# Demonstration of the Formation of a Selenocysteine Selenenic Acid through Hydrolysis of a Selenocysteine Selenenyl Iodide Utilizing a Protective Molecular Cradle

**DOI:** 10.3390/molecules28247972

**Published:** 2023-12-06

**Authors:** Kei Goto, Ryutaro Kimura, Ryosuke Masuda, Takafumi Karasaki, Shohei Sase

**Affiliations:** Department of Chemistry, School of Science, Tokyo Institute of Technology, Tokyo 152-8551, Japan

**Keywords:** selenocysteines, selenenic acids, selenenyl iodides, molecular cradles, iodothyronine deiodinase

## Abstract

Selenocysteine selenenic acids (Sec–SeOHs) and selenocysteine selenenyl iodides (Sec–SeIs) have long been recognized as crucial intermediates in the catalytic cycle of glutathione peroxidase (GPx) and iodothyronine deiodinase (Dio), respectively. However, the observation of these reactive species remained elusive until our recent study, where we successfully stabilized Sec–SeOHs and Sec–SeIs using a protective molecular cradle. Here, we report the first demonstration of the chemical transformation from a Sec–SeI to a Sec–SeOH through alkaline hydrolysis. A stable Sec–SeI derived from a selenocysteine methyl ester was synthesized using the protective cradle, and its structure was determined by crystallographic analysis. The alkaline hydrolysis of the Sec–SeI at −50 °C yielded the corresponding Sec–SeOH in an 89% NMR yield, the formation of which was further confirmed by its reaction with dimedone. The facile and nearly quantitative conversion of the Sec–SeI to the Sec–SeOH not only validates the potential involvement of this process in the catalytic mechanism of Dio, but also highlights its utility as a method for producing a Sec–SeOH.

## 1. Introduction

Selenoproteins are involved in a wide array of essential biological functions, ranging from the regulation of reactive oxygen species concentration to the biosynthesis of hormones, in which various highly reactive intermediates formed by oxidative modification of selenocysteine (Sec) residues play pivotal roles [1,2,3,4,5,6,7]. Selenocysteine selenenic acids (Sec–SeOHs) stand as prime examples of such intermediates. It is widely accepted that Sec–SeOHs, generated by the oxidation of selenocysteine selenols (Sec–SeHs), serve as key intermediates in the catalytic cycle of glutathione peroxidase (GPx), a crucial antioxidant enzyme in mammals that catalyzes the reduction of hydroperoxides by glutathione [8,9,10,11,12,13,14,15,16,17,18,19,20]. Selenocysteine selenenyl iodides (Sec–SeIs) have also garnered increasing attention as important intermediates of iodothyronine deiodinase (Dio). The Dio family controls the concentration of the active thyroid hormone (3,3′,5-triiodothyronine) through the reductive elimination of iodide from iodothyronines by Sec–SeHs in the catalytic sites, presumably leading to the formation of Sec–SeI intermediates [21,22,23,24,25,26,27,28,29,30,31,32,33,34,35].

**Figure 1:**
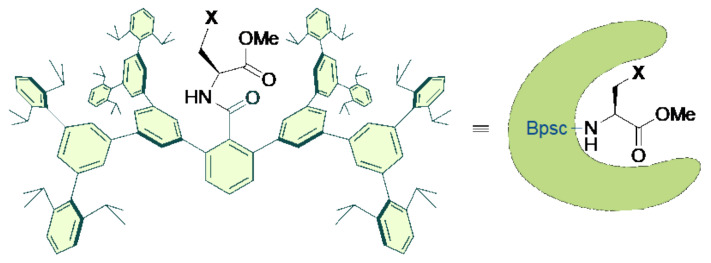
Molecular cradle for stabilization of amino acid-derived reactive species.

**Scheme 1:**
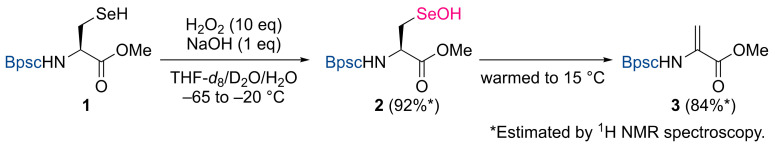
Generation of Sec–SeOH **2** by oxidation of Sec–SeH **1** and thermal deselenation of **2** to dehydroalanine **3**.

Despite the recognized importance of these Sec-derived reactive intermediates, the chemical evidence for the formation of Sec–SeOHs and Sec–SeIs remained elusive in both proteins and small-molecule systems due to their intrinsic instability. In small-molecule systems, selenenic acids undergo facile self-condensation to selenoseleninates [36], and selenenyl iodides readily disproportionate to diselenides and iodine [37,38]. As a chemical tool to stabilize such reactive species, we have developed a large molecular cradle capable of accommodating a reactive amino acid residue (Figure 1) [39,40,41,42,43]. By using the molecular cradle as an *N*-terminal protecting group (henceforth denoted as “Bpsc”), we recently succeeded in the first spectroscopic observation of Sec–SeOHs. Sec–SeOH **2** derived from a selenocysteine methyl ester was generated by the oxidation of Sec–SeH **1** with H_2_O_2_ in the presence of NaOH at −65 °C in high yield (Scheme 1) [40]. Although **2** was found to be stable at −20 °C, it underwent thermal deselenation to form the corresponding dehydroalanine **3** upon warming to 15 °C. We also achieved the synthesis and isolation of Sec–SeIs utilizing the molecular cradle [40,41,42,43,44], which were found to have high thermal stability in contrast to Sec–SeOHs.

**Scheme 2:**
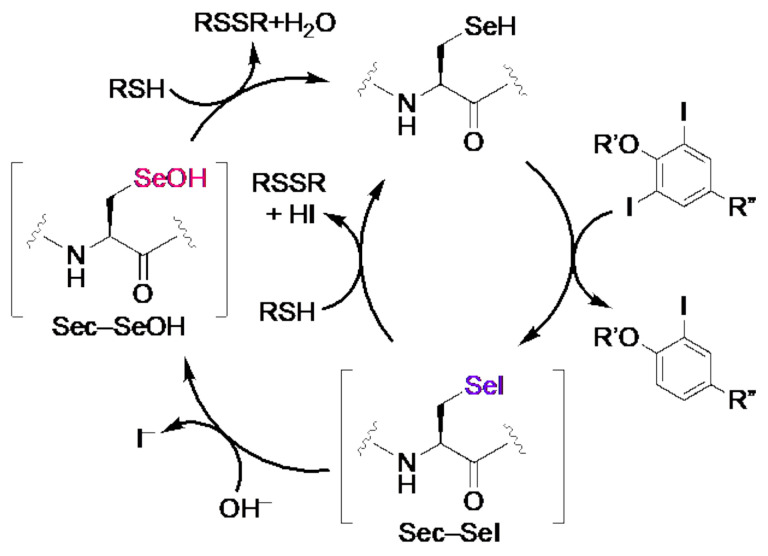
Proposed catalytic mechanism of iodothyronine deiodinase including the conversion of a Sec–SeI to a Sec–SeOH.

**Scheme 3:**
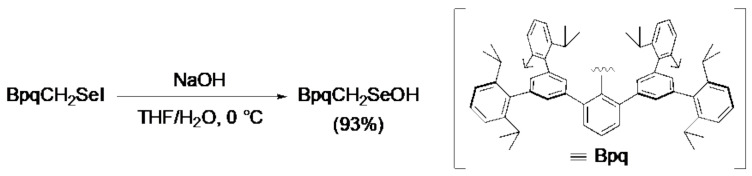
Hydrolysis of a nonselenocysteinyl derivative selenenyl iodide to form a selenenic acid.

Recently, it has been postulated that the conversion of a Sec–SeI to a Sec–SeOH is potentially involved in the Dio catalytic mechanism (Scheme 2) [27,33]. In this proposed scenario, a Sec–SeI intermediate, formed during the deiodination of iodothyronines, could rapidly hydrolyze in an aqueous environment to produce a Sec–SeOH. However, no chemical evidence has been available for this reaction process involving selenocysteine-derived reactive species both as substrate and product. We previously reported that a nonselenocysteinyl derivative selenenyl iodide bearing a bulky substituent was readily hydrolyzed to form the corresponding selenenic acid under alkaline conditions (Scheme 3) [45]. Given the ease and high yield of this reaction, it is anticipated that hydrolysis of a Sec–SeI to produce a Sec–SeOH could occur at low temperatures, where the resulting Sec–SeOH remains stable and resistant to thermal deselenation. Herein, we report the first demonstration of the chemical transformation from a Sec–SeI to a Sec–SeOH using the cradle-type model compounds.

## 2. Results and Discussion

**Figure 2:**
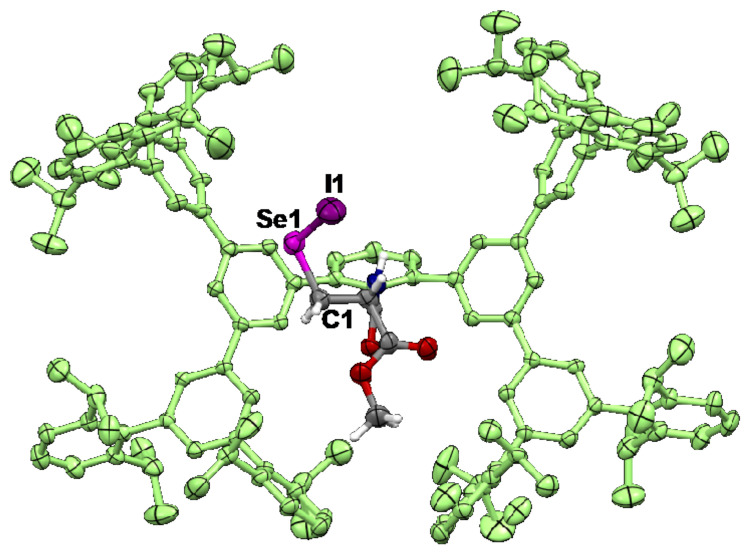
Crystal structure of Sec–SeI **4** with thermal ellipsoids drawn at the 50% probablility level. Hydrogen atoms of the Bpsc group and solvents are omitted for clarity. Selected bond lengths [Å] and bond angle [°]: I1–Se1 2.5199(10); Se1–C1 1.964(7); I1-Se1-C1 97.5(2).

**Scheme 4:**
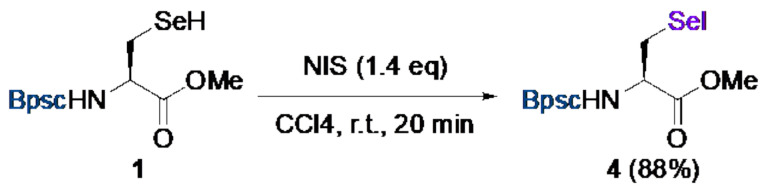
Synthesis of Sec–SeI **4** by iodination of Sec–SeH **1**.

Sec–SeI **4** derived from a selenocysteine methyl ester was synthesized by the reaction of Sec–SeH **1** [40] with *N*-iodosuccinimide (NIS) and isolated in the form of purple crystals (Scheme 4). The structure of **4** was determined by a single-crystal X-ray diffraction analysis (Figure 2). The selenocysteine moiety of **4** is accommodated in a large cavity produced by the Bpsc group (dimensions ca. 2.3 × 1.7 nm). Similar to the Sec–SeIs we previously reported [40,41,42,43,44], Sec–SeI **4** exhibited high thermal stability, with a melting point of above 250 °C. Sec–SeI **4** is also stable in air and can be manipulated as a compound having “shelf-stability”.

**Figure 3:**
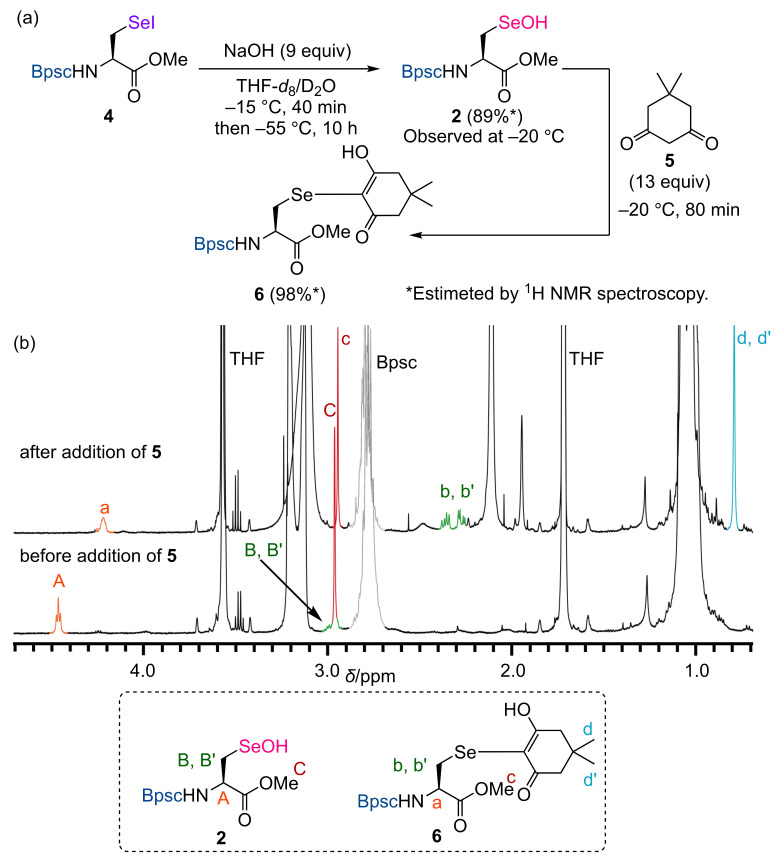
(**a**) Hydrolysis of Sec–SeI **4** to form Sec–SeOH **2** followed by the reaction with dimedone (**5**); (**b**) ^1^H NMR spectra (500 MHz, THF-*d*_8_/D_2_O) of Sec–SeOH **2** recorded at −20 °C (bottom) and selenide **6** formed by the reaction of **2** with **5** recorded at 25 °C (top).

In the catalytic mechanism of Dio, the formation of a Sec–SeI intermediate during the deiodination of iodothyronine substrates in the first half-reaction has been widely accepted (Scheme 2). However, there is no consensus on the mechanism of the second half-reaction involving iodide release and reduction of the oxidized enzyme. Among the isozymes of Dio, type-III iodothyronine deiodinase (Dio3) catalyzes the regioselective inner ring 5-deiodination. Schweizer and Steegborn suggested that a Sec–SeI intermediate generated in the active site of Dio3 might undergo the rapid exchange of iodide with hydroxide in the aqueous environment forming a Sec–SeOH intermediate [27,33]. For modeling this process, the conversion of Sec–SeI **4** to Sec–SeOH **2** through alkaline hydrolysis was investigated. The reaction was carried out at low temperatures to prevent the thermal deselenation of the resulting **2**. Sec–SeI **4** was treated with aqueous NaOH (9 equiv) in THF-*d*_8_/D_2_O at −15 °C (Figure 3a). The mixture was stirred at the same temperature for 40 min and then at −50 °C for 10 h, during which the purple solution turned colorless, indicating the consumption of Sec–SeI **4**. A portion of the resulting solution was transferred to a J-Young NMR tube via a tube cooled to −55 °C, and ^1^H NMR spectrum was recorded at −20 °C. The formation of Sec–SeOH **2** in 89% NMR yield was observed in the ^1^H NMR spectrum (Figure 3b and Figure S4), while only a slight amount of dehydroalanine **3** was detected (Scheme S1). The observed signals of Sec–SeOH **2** matched those of **2** generated by the H_2_O_2_ oxidation of Sec–SeH **1** (Scheme 1) [40]. The identification of the main product as **2** was further confirmed by the reaction with dimedone (**5**). After treatment with dimedone (**5**) (13 equiv) at −20 °C for 80 min, Sec–SeOH **2** was converted to selenide **6** almost quantitatively (Figure 3a,b), which is similar to the reaction of **2** generated by H_2_O_2_ oxidation of **1** to form **6** [40]. Thus, by harnessing the protective molecular cradle, the chemical transformation from a Sec–SeI to a Sec–SeOH has been experimentally demonstrated for the first time, corroborating the chemical validity of the proposal that the hydrolytic conversion of a Sec–SeI to a Sec–SeOH is potentially involved in the catalytic mechanism of Dio3. This reaction process represents an interconversion of the oxidized forms of selenoproteins at the same oxidation level, suggesting the necessity of careful consideration regarding which oxidized form is engaged in enzymatic reactions.

In the synthesis of Sec–SeOH **2** through the oxidation of Sec–SeH **1** with H_2_O_2_, an excess of oxidant was required to enhance the conversion efficiency to **2** (Scheme 1) [40]. In contrast, the formation of Sec–SeOH **2** through the hydrolysis of Sec–SeI **1** proceeded in high yield under mild conditions, without involving any redox processes, highlighting the utility of this reaction as a valuable, cleaner method for producing a Sec–SeOH.

## 3. Materials and Methods

### 3.1. General

CCl_4_ was purchased from commercial sources and dried over 4ÅMS. CDCl_3_ was passed through a small column of neutral alumina prior to use. In the NMR experiments, THF-*d*_8_ was purchased from commercial sources, distilled over CaH_2_, and degassed through freeze-pump-thaw cycles. Other chemicals were purchased from commercial sources and used as received. ^1^H NMR spectra were recorded on a JEOL ECX-500, a JEOL ECZ-500, and the chemical shifts of ^1^H are referenced to the residual proton signal of CDCl_3_ (δ 7.25) and THF-*d*_8_ (1.72). ^13^C NMR spectrum was recorded on a JEOL ECX-500, and the chemical shifts are given relative to CDCl_3_ (δ 77.00) as an internal standard. ^77^Se NMR spectrum was recorded on a JEOL ECX-500, and the chemical shifts of ^77^Se are referenced to Ph_2_Se_2_ (δ 480) as an external standard. All spectra were assigned with the aid of DEPT, COSY, HMQC, and HMBC NMR experiments. IR spectrum was recorded on a JASCO FT/IR-4100. UV-vis spectrum was recorded on a JASCO V-650 UV-vis spectrometer. A high-resolution FD-TOF mass spectrum was measured on a JEOL JMS-T100GCv “AccuTOF GCv”. Melting points (m.p.) were measured with a Yanaco MP-S3 (uncorrected).

### 3.2. Synthesis of Sec–SeI ***4***

Sec–SeH **1** (173 mg, 86.4 μmol), which was prepared by the reported procedure [40], was placed in a 25 mL Schlenk tube. After being evacuated and backfilled with argon, degassed CCl_4_ (8.5 mL) and then NIS (27.2 mg, 0.121 mmol) were added. The resulting reaction mixture was stirred at room temperature for 20 min. The resulting purple solution was filtered through Celite and then concentrated in vacuo. The obtained crude product was recrystallized from Et_2_O-pentane to give Sec–SeI **4** as purple crystals. Yield 164 mg (76.3 μmol, 88%).

**4:** purple crystals; m.p. 251–253 °C. ^1^H NMR (500 MHz, CDCl_3_): δ 1.03–1.05 (m, 48H), 1.10–1.14 (m, 48H), 2.70–2.79 (m, 17H), 3.03–3.06 (m, 4H), 4.58 (dt, *J* = 7.4, 3.7 Hz, 1H), 6.51 (d, *J* = 7.4 Hz, 1H), 6.99 (br, 4H, E1), 7.17–7.19 (m, 16H), 7.32 (t, *J* = 7.7 Hz, 8H), 7.43 (d, *J =* 1.4 Hz, 8H), 7.43–7.45 (m, 2H), 7.48–7.51 (m, 1H), 7.68 (d, *J* = 1.4 Hz, 4H), 7.81 (br, 2H); ^13^C NMR (125 MHz, CDCl_3_): δ 24.2 (q), 24.3 (q), 29.8 (t), 30.37 (d), 30.39 (d), 51.3 (d), 52.0 (q), 122.5 (d), 126.0 (d), 126.4 (d), 127.0 (d), 127.9 (d), 129.4 (d), 129.9 (d), 130.3 (d), 134.3 (s), 139.0 (s), 139.9 (s), 140.2 (s), 141.0 (s), 141.4 (s), 141.76 (s), 146.7 (s), 167.8 (s), 168.9 (s); ^77^Se NMR (95 MHz, CDCl_3_): δ 392; IR (KBr); 3415, 3060, 3033, 2961, 2926, 2867, 1749, 1681, 1578, 1490, 1461, 1382, 1361, 1325, 1260, 1250, 873, 805, 754 cm^−1^; UV-vis (CHCl_3_, 298 K) *λ*_max_ 496 nm (*ε* = 78); HRMS (FD-TOF) *m*/*z* 2150.0944 [M]^+^ (calcd for C_143_H_164_INO_3_Se, 2150.0921). Anal. calcd. for C_143_H_164_INO_3_Se: C, 78.86; H, 7.69; N, 0.65; found: C, 78.91; H, 7.50; N, 0.69%. For the ^1^H, ^13^C, and ^77^Se NMR spectra, see Supplementary Materials.

### 3.3. Hydrolysis of Sec–SeI ***4*** and Derivatization of Resulting Sec–SeOH ***2*** with Dimedone (***5***)

All solvents were degassed by argon bubbling before use. A stock solution of NaOH (93% purity, 92.3 mg, 2.15 mmol) in D_2_O (0.50 mL) was prepared prior to the reaction. Sec–SeI **4** (23.0 mg, 11.4 μmol) was placed in a 10 mL J-Young tube. After being evacuated and backfilled with argon, THF-*d*_8_ (1.2 mL), 1 μL of bis(trimethylsilyl)methane as an internal standard, and then a stock solution of NaOH (4.3 M, 23 μL, 99 μmol) were added at −15 °C. The mixture was stirred at the same temperature for 40 min and then stirred at −50 °C for 10 h. A portion (0.50 mL) of the resulting colorless solution was transferred to a J-Young NMR tube via cooled tube (−55 °C) carefully. A ^1^H NMR spectrum was recorded at −20 °C. The formation of Sec−SeOH **2** was observed in 89% NMR yield (Figures S4 and S5a). Dimedone (**5**; 8.2 mg, 58 μmol) was then added to the sample in J-Young NMR tube at −55 °C. After standing at −20 °C for 80 min, the ^1^H NMR spectrum was recorded at room temperature. The formation of selenide **6** was observed in 87% NMR yield, while **2** was not detected (Figure S5b). The ^1^H NMR signals of **2** and **6** observed in the above experiments matched those we previously reported [40].

### 3.4. X-ray Crystallographic Analysis of Sec–SeI ***4***

Single crystals of **4**∙C_4_H_10_O∙0.5C_5_H_12_ were grown in their Et_2_O-pentane solution. A purple crystal of **4**∙C_4_H_10_O∙0.5C_5_H_12_ was mounted on a loop. The measurement was made on a Rigaku/Synergy CCD with VariMax Mo with graphite monochromated Mo-Kα radiation (λ = 0.71075 Å) at –150 °C. The structures were solved and refined against all F2 values using Shelx-2018 [46] implemented through Olex2 v1.3. The non-hydrogen atoms were refined anisotropically, except for the minor components of the disordered isopropyl groups. The hydrogen atoms were idealized by using the riding models. An attempt to sensibly model the solvent molecules (probably eight pentanes which was used for final crystallization) was unsuccessful because of the diffuse electron density (disordered) corresponding to them and limited data quality. So, the solvent mask (similar to PLATON_SQUEEZE) was applied using Olex2 to remove those electron densities in the final model. The solvent accessible volume was found to be 3958.9 Å^3^ and the number of the electrons found in a solvent accessible void is 665.9 e^−^, which corresponds to approximately four pentane molecules per unit cell. The slight variation in void electron count can be the result of limited data quality. CCDC 2303923 contains the supplementary crystallographic data for this paper. These data can be obtained free of charge via http://www.ccdc.cam.ac.uk/data_request/cif accessed on 10 November 2023 (or from the CCDC, 12 Union Road, Cambridge CB2 1EZ, UK; Fax: +44 1223 336033; E-mail: deposit@ccdc.cam.ac.uk).

## 4. Conclusions

The chemical transformation from a Sec–SeI to a Sec–SeOH was demonstrated for the first time using the cradle-type model compounds. The alkaline hydrolysis of a Sec–SeI derived from a selenocysteine methyl ester proceeded at low temperatures to produce the corresponding Sec–SeOH almost quantitatively. These findings not only imply the potential relevance of this conversion process in enzymatic function, but also underscore its utility as a synthetic approach for producing a Sec–SeOH. Further investigations on the detailed mechanisms of the Dio second half-reaction from a Sec–SeI or a Sec–SeOH to a Sec–SeH are currently underway, utilizing selenopeptide model systems that mimic the catalytic site of Dio.

## Data Availability

The data presented in this study are available on request from the corresponding author.

## Supplementary Materials

The following supporting information can be downloaded at: https://www.mdpi.com/article/10.3390/molecules28247972/s1, Figures S1–S5: NMR spectra; Figure S6: Molecular structure; Scheme S1: Detailed reaction scheme; Table S1: Crystallographic data.
