# Supplementary material for: Demonstration of the Formation of a Selenocysteine Selenenic Acid through Hydrolysis of a Selenocysteine Selenenyl Iodide Utilizing a Protective Molecular Cradle
Source: Molecules. 2023 Dec 6;28(24):7972. doi: 10.3390/molecules28247972 (PMC10746021; doi:10.3390/molecules28247972)
Supplement: Supplementary file 1 [file molecules-28-07972-s001.zip › molecules-2725463-supplementary.pdf]

Supplementary Materials for:

Demonstration of the Formation of a Selenocysteine Selenenic Acid through Hydrolysis of a Selenocysteine Selenenyl Iodide Utilizing a Protective Molecular Cradle

Kei Goto,* Ryutaro Kimura, Ryosuke Masuda, Takafumi Karasaki, and Shohei Sase
Department of Chemistry, School of Science, Tokyo Institute of Technology
*Correspondence: goto@chem.titech.ac.jp

Contents

NMR spectra of Sec–SeI **4** S2
Hydrolysis of Sec–SeI **4** and derivatization of resulting Sec–SeOH **2** with dimedone (**5**) S4
X-ray crystallographic analysis of Sec–SeI **4** S6
References S7

NMR spectra of Sec–SeI 4

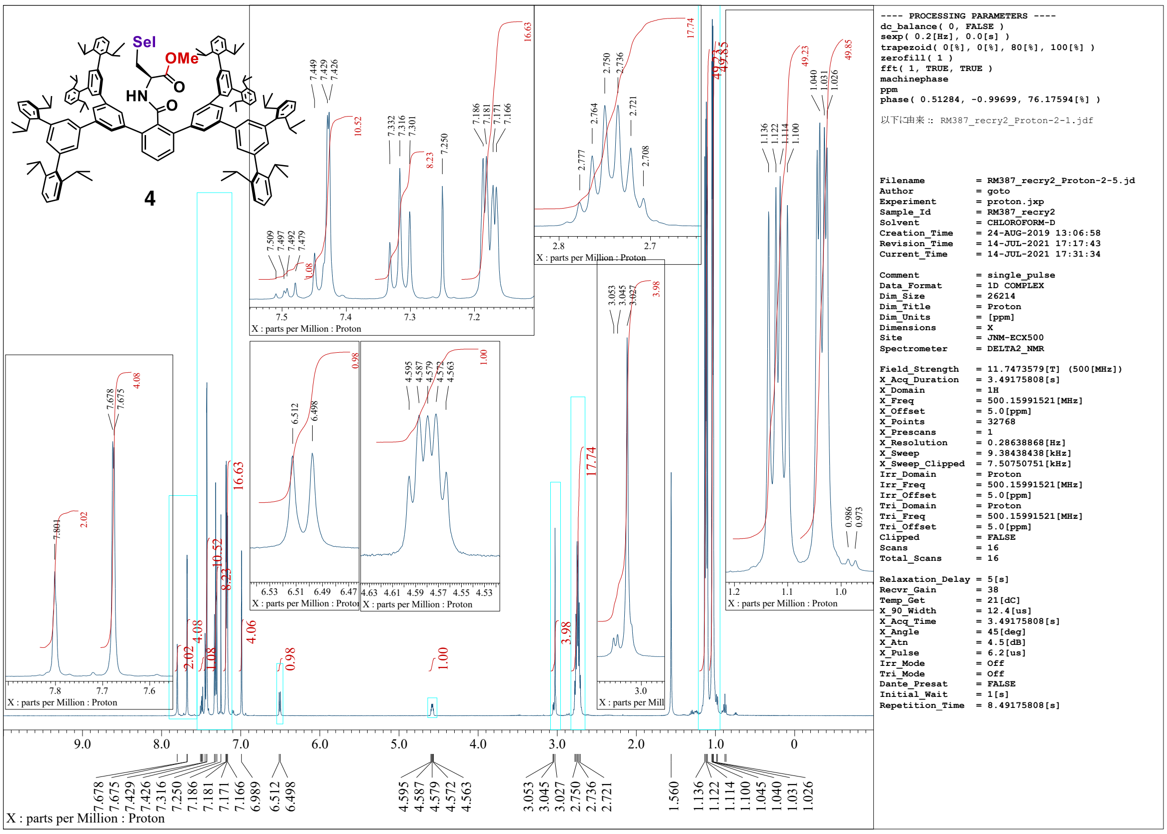

Figure S1. ^{1}H NMR (500 MHz, $CDCl_3$) spectrum of **4**.

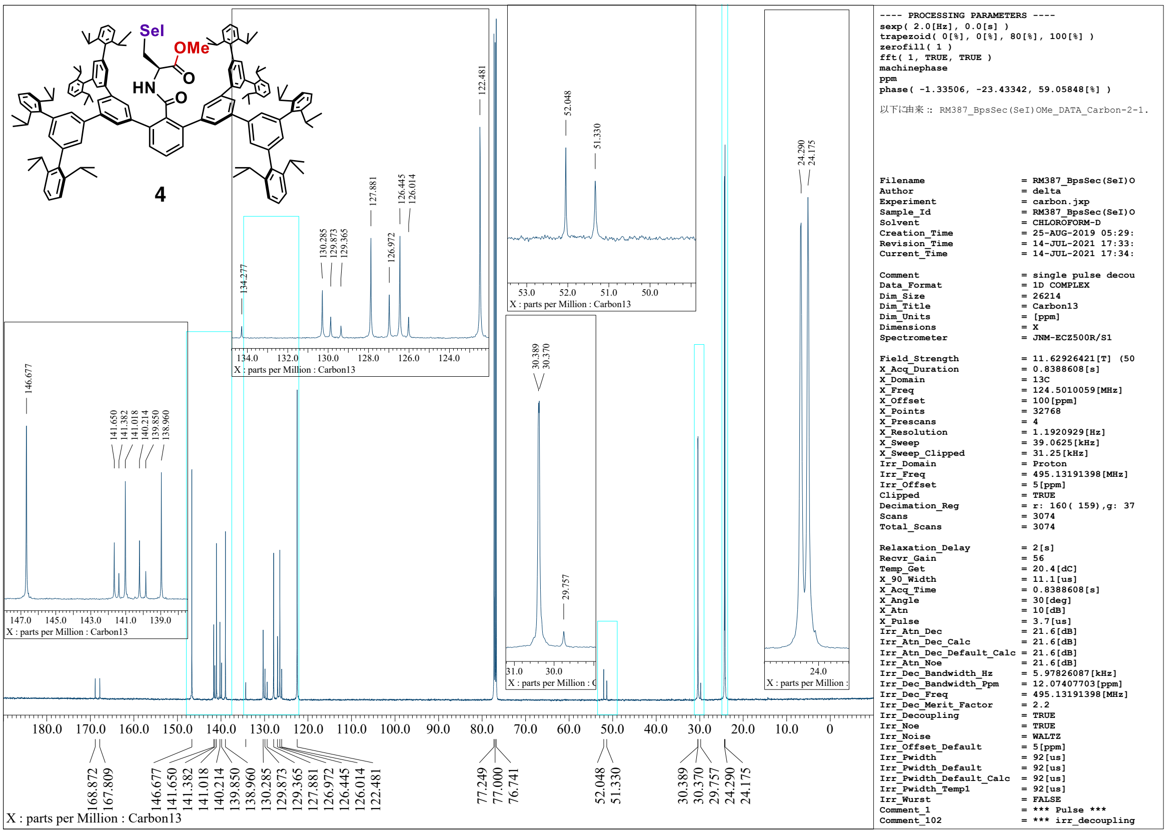

Figure S2. ^{13}C NMR (125 MHz, $CDCl_3$) spectrum of **4**.

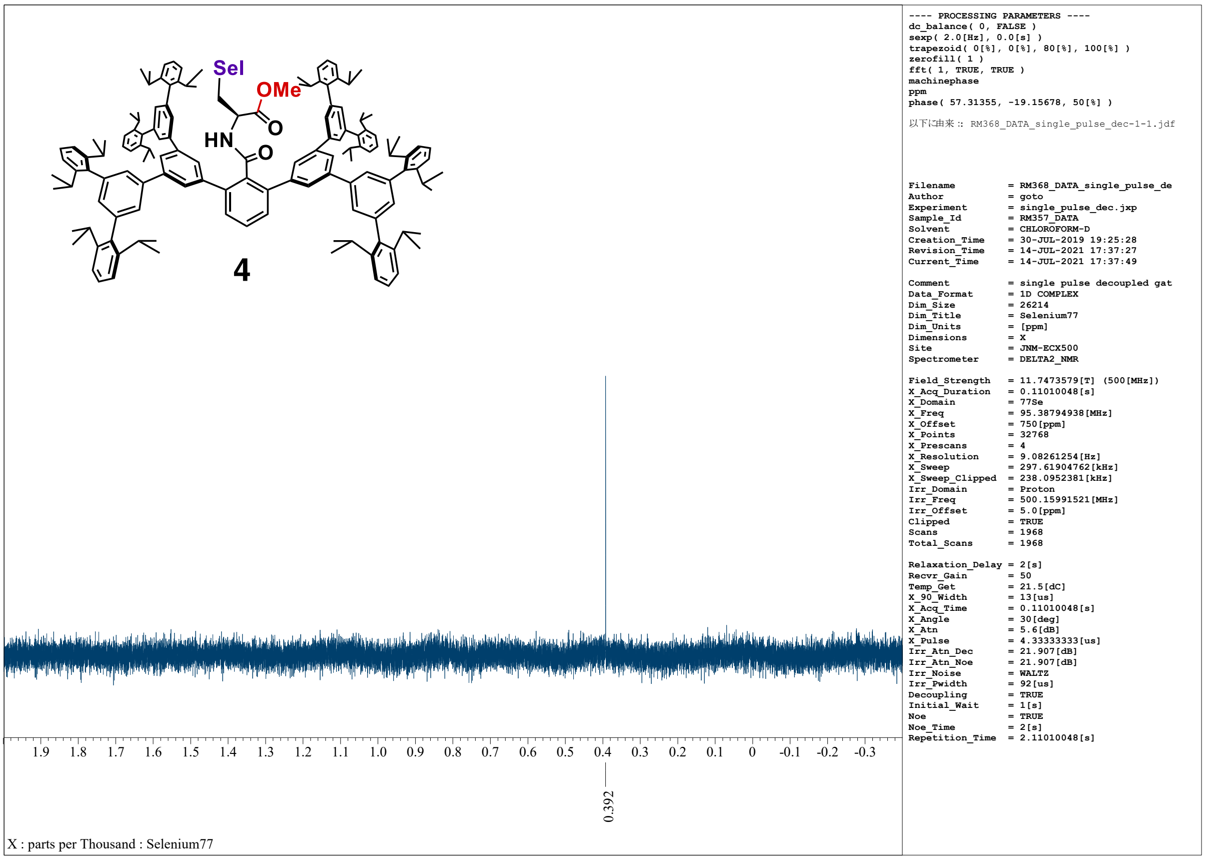

Figure S3. ^{77}Se NMR (95 MHz, $CDCl_3$) spectrum of **4**.

Assignment of ^{1}H and ^{13}C NMR spectra

4: ^{1}H NMR (500 MHz, $CDCl_3$): δ 1.03-1.05 (m, 48H, **C** and **D**), 1.10-1.14 (m, 48H, **C** and **D**), 2.70-2.79 (m, 17H, **B** and **K$_A$**), 3.03-3.06 (m, 4H, **M** and **K$_B$**), 4.58 (dt, J = 7.4, 3.7 Hz, 1H, **J**), 6.51 (d, J = 7.4 Hz, 1H, **I**), 6.99 (br, 4H, **E1**), 7.17-7.19 (m, 16H, **A2**), 7.32 (t, J = 7.7 Hz, 8H, **A1**), 7.43 (d, J = 1.4 Hz, 8H, **E3**), 7.43-7.45 (m, 2H, **G3**), 7.48-7.51 (m, 1H, **G4**), 7.68 (d, J = 1.4 Hz, 4H, **F3**), 7.81 (br, 2H, **F1**); ^{13}C{^{1}H} NMR (125 MHz, $CDCl_3$): δ 24.2 (q, CH($\underline{C}H_3)_2$), 24.3 (q, CH($\underline{C}H_3)_2$), 29.8 (t, **K**), 30.37 (d, $\underline{C}H(CH_3)_2$), 30.39 (d, $\underline{C}H(CH_3)_2$), 51.3 (d, **J**), 52.0 (q, **M**), 122.5 (d, **A2**), 126.0 (d, **F1**), 126.4 (d, **E3**), 127.0 (d, **F3**), 127.9 (d, **A1**), 129.4 (d, **G4**), 129.9 (d, **G3**), 130.3 (d, **E1**), 134.3 (s, **G1**), 139.0 (s, **A4**), 139.9 (s, **G2**), 140.2 (s, **F2**), 141.0 (s, **E2**), 141.4 (s, **F4**), 141.76 (s, **E4**), 146.7 (s, **A3**), 167.8 (s, **H**), 168.9 (s, **L**).

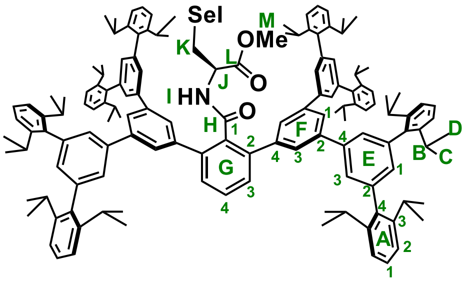

Hydrolysis of Sec–SeI 4 and derivatization of resulting Sec–SeOH 2 with dimedone (5)

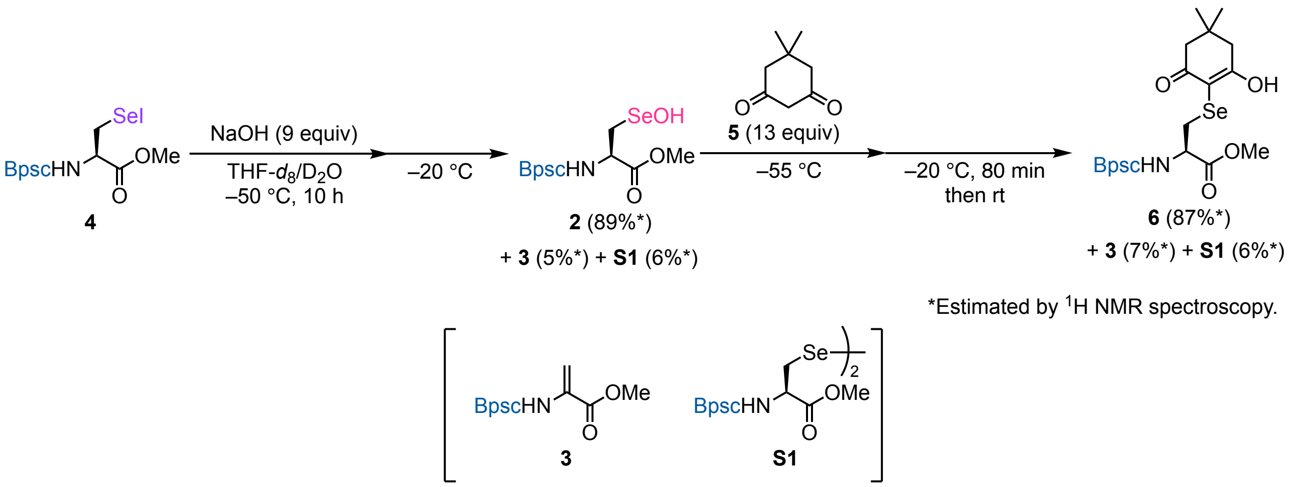

Scheme S1. Hydrolysis of Sec–SeI **4** and derivatization of resulting Sec–SeOH **2** with dimedone (**5**).

All solvents were degassed by argon bubbling before use. A stock solution of NaOH (93% purity, 92.3 mg, 2.15 mmol) in D_2O (0.50 mL) was prepared prior to the reaction. Sec–SeI **4** (23.0 mg, 11.4 µmol) was placed in a 10 mL J-Young tube. After evacuated and backfilled with argon, THF-d_8 (1.2 mL), 1 µL of bis(trimethylsilyl)methane as an internal standard, and then a stock solution of NaOH (4.3 M, 23 µL, 99 µmol) were added at −15 °C. The mixture was stirred at the same temperature for 40 min and then stirred at −50 °C for 10 h. 0.50 mL of the obtained colorless solution was transferred to a J-Young NMR tube via cooled tube (−55 °C) carefully. A ^{1}H NMR spectrum was recorded at −20 °C. The formation of Sec−SeOH **2**, dehydroalanine **3**, and diselenide **S1**[1] were observed in 89%, 5%, 6% NMR yields, respectively (Figure S4, Figure S5a). Then, dimedone (**5**; 8.2 mg, 58 µmol) was added to the sample in J-Young NMR tube at −55 °C. After 80 min, the ^{1}H NMR spectrum was recorded at room temperature. The formation of selenide **6**,[1] **3**, and **S1** were observed in 87%, 7%, 6% NMR yields, respectively, while Sec–SeOH **2** was not detected (Figure S5b).

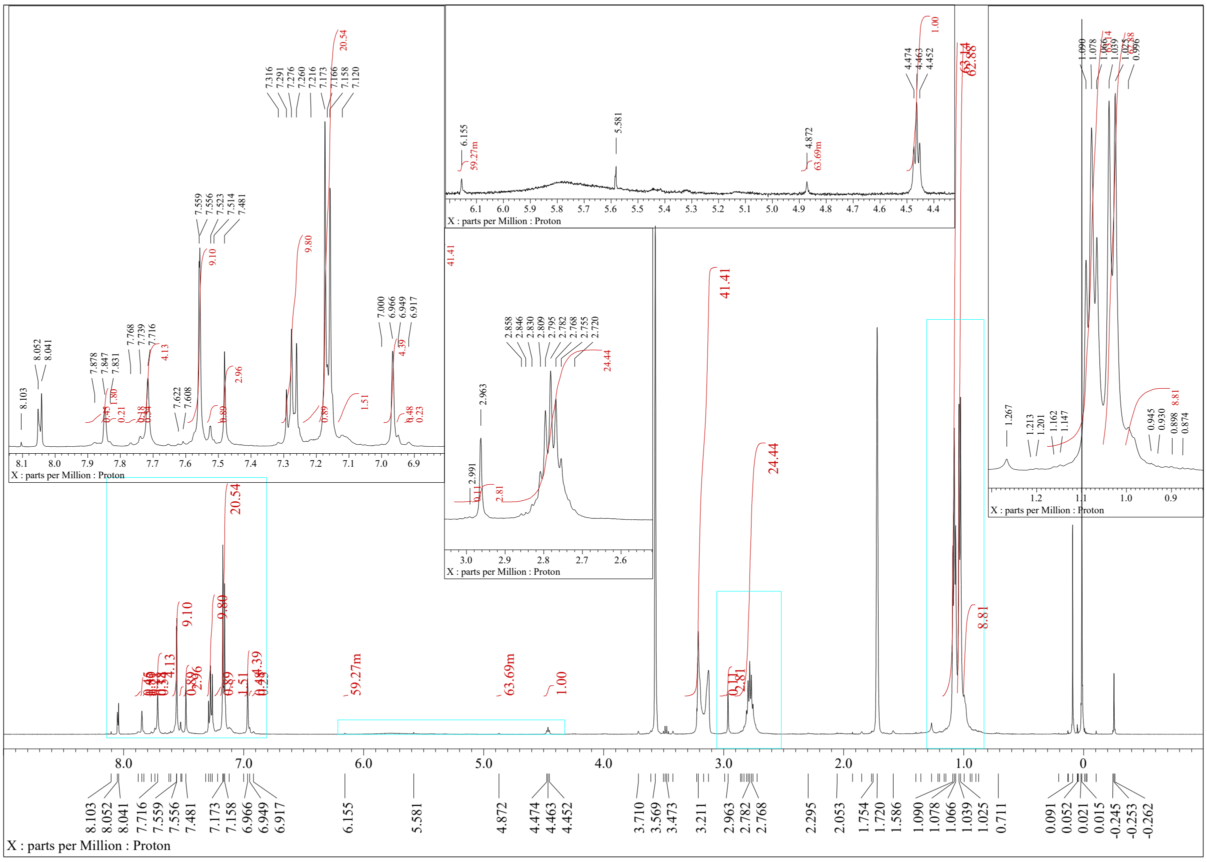

Figure S4. ^{1}H NMR (500 MHz, THF-d_8/D_2O, −20 °C) spectrum of the mixture obtained by the reaction of **4** with NaOH in Scheme S1.

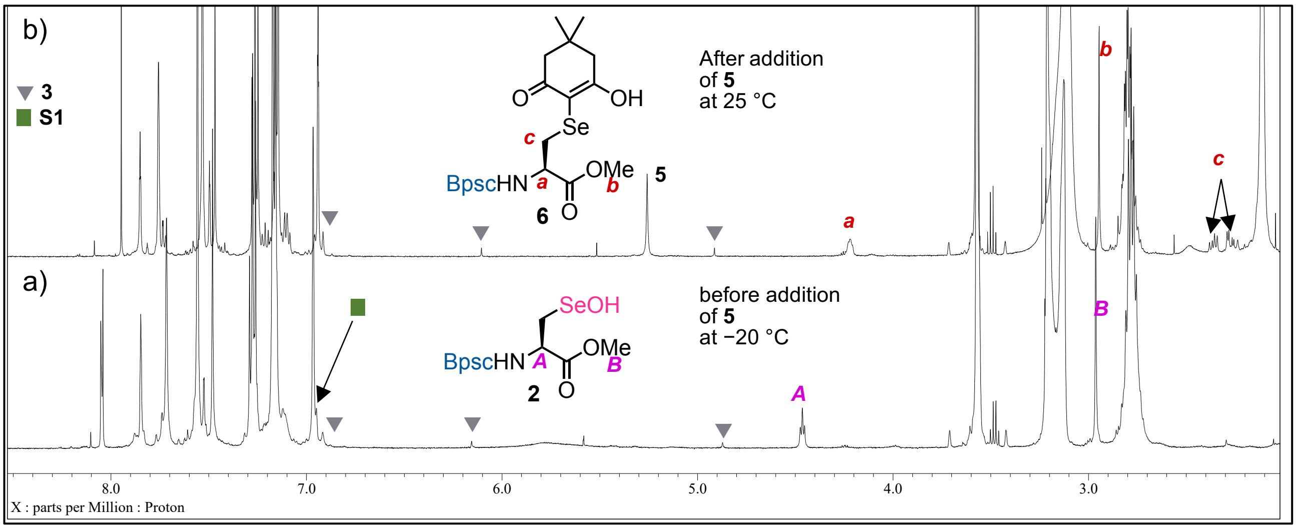

Figure S5. ^{1}H NMR (500 MHz, THF-d_8/D_2O) spectra of (a) **2** at −20 °C and (b) after addition of **5** at 25 °C in Scheme S1.

X-ray crystallographic analysis of Sec–SeI 4

Single crystals of **4**·$C_4H_{10}O$·0.5C_5H_{12} were grown in their Et_2O-pentane solution. A purple crystal of **4**·$C_4H_{10}O$·0.5C_5H_{12} was mounted on a loop. The measurement was made on a Rigaku/Synergy CCD with VariMax Mo with graphite monochromated Mo-Kα radiation (λ = 0.71075 Å) at –150 °C. Crystallographic and experimental data are listed in Table S1. The structures were solved and refined against all F^2 values using Shelx-2018[2] implemented through Olex2 v1.3. The non-hydrogen atoms were refined anisotropically, except for the minor components of the disordered isopropyl groups. The hydrogen atoms were idealized by using the riding models. Attempt to sensibly model the solvent molecules (probably four pentanes which was used for final crystallization) was unsuccessful because of diffuse electron density (disordered) corresponding to them and limited data quality. So, the solvent mask (similar to PLATON_SQUEEZE) was applied using Olex2 to remove those electron densities in the final model. The solvent accessible volume was found to be 3958.9 Å^3 and the number of the electrons found in solvent accessible void is 665.9 e$^-$, which correspond to approximately four pentane molecules per unit cell. The slight variation in void electron count can be the result of limited data quality.

Table S1. Crystal data and structure refinement for **4**·$C_4H_{10}O$·0.5C_5H_{12}.

Empirical formula	$C_{143}H_{164}INO_3Se$·$C_4H_{10}O$·0.5C_5H_{12}	
Formula weight	2260.79	
Temperature	123.35 K	
wavelength	0.71075 Å	
Crystal system	orthorhombic	
Space group	$P2_12_12_1$	
Unit cell dimensions	a = 13.5401(15) Å	α = 90°
	b = 27.601(3) Å	β = 90°
	c = 42.365(5) Å	γ = 90°
Volume	15833(3) Å^3	
Z	4	
D_{calc}	0.948 g/cm^3	
Absorption coefficient	0.475 mm^{-1}	
F(000)	4812.0	
Crystal size	0.22 x 0.17 x 0.07 mm^3	
Theta range for data collection	3.0090 to 27.4584°	
Index ranges	$-9 \le h \le 17$, $-35 \le k \le 35$, $-45 \le l \le 53$	
Reflections collected	58743	
Independent reflections	33824 [R_{int} = 0.0462]	
Max. and min. transmission	0.771 and 1.000	
Completeness to theta = 50.5°	99.5%	
data / restraints / parameters	33824 / 63 / 1474	

Goodness-of-fit on F^2	0.980
Final R induces (I>2σ(I))	R_1 = 0.0790, wR_2 = 0.1969
R induces (all data)	R_1 = 0.1046, wR_2 = 0.2223
Largest diff. peak and hole	0.96 and −1.35 e. Å^{-3}
Flack parameter	–0.016(6)

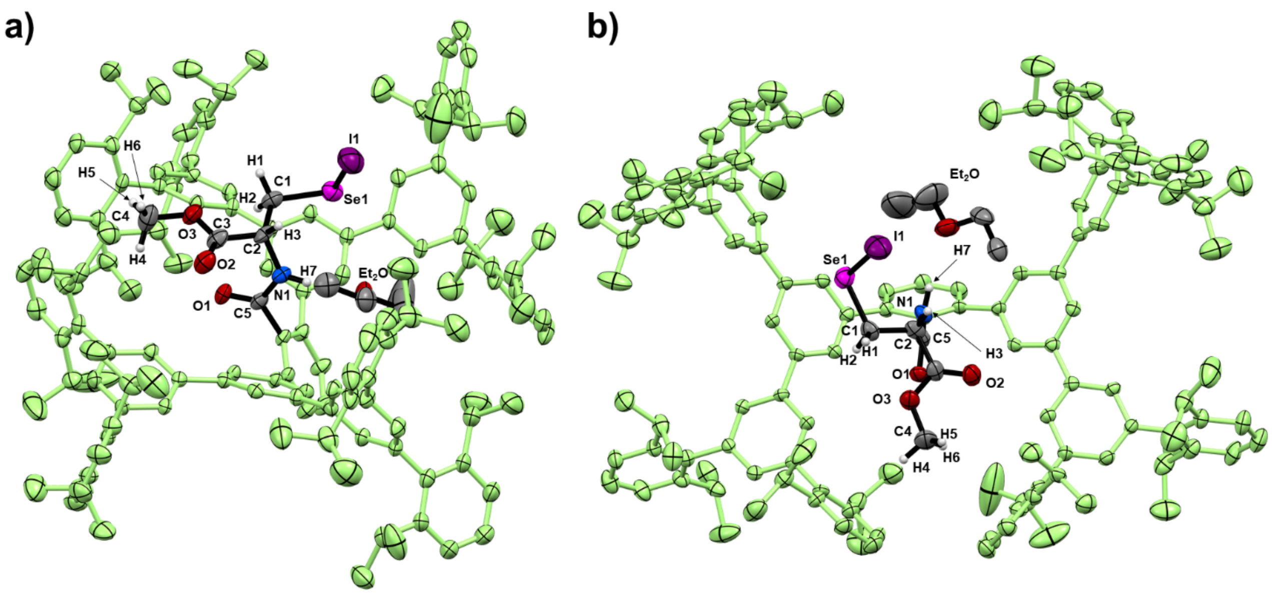

Figure S6. Molecular structure of **4** with thermal ellipsoids at 50% probability: (a) Front view, and (b) top view. Only the position with higher occupancy of the disordered isopropyl group in the Bpsc group is shown. Hydrogen atoms of the Bpsc groups and solvents are omitted for clarity.

References

1. (a) Masuda, R.; Kimura, R.; Karasaki, T.; Sase, S.; Goto, K. *J. Am. Chem. Soc.* **2021**, *143*, 6345–6350. (b) Masuda, R.; Goto, K. *Methods Enzymol.* **2022**, *662*, 331–361. (c) Masuda, R.; Kuwano, S.; Sase, S.; Bortoli, M.; Madabeni, A.; Orian, L.; Goto, K. *Bull. Chem. Soc. Jpn.* **2022**, *95*, 1360–1379. (d) Masuda, R.; Takafumi, K.; Sase, S.; Kuwano, S.; Goto, K. *Chem. - Eur. J.* **2023**, e202302615.

2. Sheldrick, G. M. *Acta Crystallogr., Sect. C: Struct. Chem.* **2015**, *C71*, 3–8.
